# A role for features in speech perception

**DOI:** 10.1177/02676583251334518

**Published:** 2025-07-16

**Authors:** Heather Goad

**Affiliations:** McGill University, Canada

**Keywords:** speech perception, features, abstractness

## Abstract

Archibald’s article makes a strong case for abstract symbolic representations in the phonological grammars of second language learners/users (L2ers). The evidence he brings to bear on this comes principally from the prosodic domain. However, the case for abstractness is hardest to defend in the segmental domain, specifically when it comes to motivating a role for features. The goal of this commentary is to show that L2 speech perception is mediated, in part, by features and thereby provide support for Archibald’s claim. Two speech perception studies are discussed. The first study shows that the status of the feature [nasal] in vowels in the first language (L1) grammar, as contrastive (French) or allophonic (English), impacts naive perception of novel nasal vowels. French listeners successfully perceive the novel vowels; English listeners’ success is hindered by the phonological status of [nasal] in the L1 grammar. The results are proposed to support a role for abstract phonological representations: for features; for the conditions under which they must be shared across segments; and for a theory of licensing that can capture the licensing potential of different prosodic positions. The second study shows that the absence of the feature [SG] from the L1 grammar of French negatively impacts the ability to perceive and build an appropriate representation for English /h/. The lack of [SG] is proposed to account for three types of behaviour displayed by L2ers: their failure to perceive [h] as distinct from Ø; their VOT values for ‘voiced’ and ‘voiceless’ stops which fall between those of L1 French and those of target L2 English; and their overapplication of aspiration to stops in sC clusters. It is shown that these patterns cohere under a phonological account, as features have a classificatory function and are thereby expected to shape phonological behaviour across multiple groups of segments.

## I Introduction

John Archibald’s thought-provoking article, ‘Waiting in the wings: The place of phonology in the study of multilingual grammars’ (Archibald, 2025), makes a strong case for abstract symbolic representations in all components of the grammar. Phonology is, in this way, claimed to be no different from syntax. However, a growing body of work in phonology has largely set abstractness aside. The earlier held view championed by Chomsky and Halle (1968) that phonetics, which is grounded in constraints on articulation and acoustics, interprets the output of phonology, which manipulates abstract categorical representations, is difficult to defend in the face of evidence that phonological patterns are gradient (Cohn, 2006; Pierrehumbert et al., 2000) and that phonetically motivated constraints shape phonological behaviour (Prince and Smolensky, 2004).

Even if one advocates for abstract representations in phonological theory, it does not necessarily follow that the grammars of second language learners/users (henceforth L2ers) employ these same types of representations. L2 grammars could be fundamentally different from those of native speakers and, thus, Archibald’s article largely focuses on the question: ‘Are abstract symbolic constituents invoked in the phonological knowledge of [L2ers]?’ Archibald answers this question in the affirmative, but the body of evidence he brings to bear on this question comes principally from the prosodic domain, including L2ers’ patterns in the acquisition of syllable structure, stress, phrasing and prosodic morphology. Although native speaker grammars exhibit gradience in the prosodic domain, for example, onset profile can perturb the location of stress (e.g. Ryan, 2014), the case for categorical representations is hardest to defend in the segmental domain, specifically, when it comes to motivating a role for features.

Although features are conventionally assumed to be phonetically grounded (Chomsky and Halle, 1968; Jakobson et al., 1952; see also Mielke, 2008), there is no one-to-one relation between articulatory targets or acoustic cues and the featural specification of segments. Beyond its phonetic attributes, the presence of a feature is determined by its need to capture contrast and express phonological behaviour (e.g. assimilation rules, cooccurrence constraints), which means that the same segment can be represented differently across languages (Abaglo and Archangeli, 1989; Dresher, 2009).

If the appropriate featural representations for a language L are entwined with the phonological processes that characterize L, we might expect these representations to be inaccessible or irrelevant to the speech perception patterns displayed by native speakers of L when they are exposed to a new language. In other words, L2 speech perception could be driven entirely by the acoustic profile of first language (L1) and L2 segments in context. Indeed, Archibald points out that: ‘Work within cross-linguistic models of production and/or perception . . ., for the most part, is concerned with the properties of the physical systems (i.e. phonetics) rather than how the sounds are connected to meaning in a mental grammar.’

In view of this, my overall goal is to show that L2 perception is mediated – at least in part – by features and thereby provide support for Archibald’s claim that abstract symbolic representations underlie L2ers’ phonological behaviour at the sub-segmental level. I report on two speech perception studies that support this claim. The first shows that the status of a feature in the L1 grammar, as contrastive or allophonic, can impact L2 perception of novel vowels that require that feature; the second shows that the absence of a feature from the L1 grammar can negatively impact the ability to perceive and build novel segments in the L2.

## II When can features be redeployed?

It goes without saying that in all versions of feature theory, the presence of contrast determines that the features implicated are present in the lexical entries of morphemes and can be manipulated by the grammar.^1^ Beyond contrast, there is less agreement: non-contrastive features may have the same formal status as contrastive features because they too are involved in phonological rules and constraints that shape surface forms; alternatively, non-contrastive features may be assigned a secondary role, given that the rules that act on them yield outputs that are not structure preserving (Kiparsky, 1985).

Feature-based approaches to L2 acquisition assign a central role to contrast. Brown (1998) proposes that L2ers can perceive non-native segments only when the features needed to capture these distinctions are contrastively present in the L1 grammar. In the terminology of Archibald (2005), this can be expressed as a strong version of the Feature Redeployment Hypothesis: only features that are contrastive in the L1 grammar can be recombined to facilitate perception of novel segments in the L2. Although Brown’s position has been shown to be too strong (for more nuanced definitions of Redeployment, see, for example, Archibald, 2005, 2023), we will see that it appropriately characterizes the behaviour of naive learners which, in turn, could optimally position learners from certain L1 backgrounds for early success in real language learning.

The first study we consider is Martinez et al.’s (2023) exploration of redeployment of the feature [nasal] by naive learners of the Brazilian Portuguese high front vowel contrast, /i/ vs. /ĩ/. We focus on three groups of learners from their study: French, English and non-Caribbean Spanish (speakers of Mexican, South American and most Peninsular varieties). The status of nasality in vowels in these three languages differs: in French, it is phonemic (contrastive) for non-high vowels; in English, it is allophonic (assigned by rule); and in Spanish, it is phonetic (involves coarticulation).

Earlier research reports that the implementation of nasality for vowels in languages where it is phonemic or allophonic is intended and controlled by speakers and, as a result, even in the case of allophonic nasalization, it extends some distance back into the vowel (Moraes, 1997; Solé, 1992). In contrast, in languages where nasalization is phonetic, it is unintended, automatic and short in duration reflecting coarticulation (Solé, 1992). Martinez et al. interpret these differences as follows: in the grammars of French and English, the feature [nasal] is specified in vowels; it can express contrasts when phonemic and can be involved in phonological operations when allophonic or phonemic. In contrast, in the grammar of Spanish, [nasal] is not phonologically present in vowels.

Using an AXB discrimination task, Martinez et al. observe that both French and English listeners performed significantly higher than chance on [i]–[ĩ], whereas Spanish listeners did not. From a featural point of view, this suggests that [nasal] can be redeployed, whether it plays a phonemic or allophonic role in the L1 grammar. From a perceptual point of view, the same results would likely be expected, as the articulatory findings for phonemic and allophonic nasalization described above suggest that, in both French and English, but not in Spanish, a minimum threshold for nasality in vowels in the L1 grammar has been reached or surpassed to ensure perceptual success of nasality in the L2.

However, it seems that the featural and perceptual accounts diverge when the results of an additional contrast examined by Martinez et al. are considered, which reveal that the phonological status of the feature in vowels, phonemic vs. allophonic, and not the extent of nasality present on vowels in the L1, is predictive. Specifically, listeners were tested on their ability to discriminate [ĩ]–[iŋ]. The results for this contrast, alongside [i]–[ĩ], are shown in Table 1.

**Table 1.** Perception of contrasts across three groups of listeners.

	Contrast perceived as:		
Contrast tested	French	English	Spanish
[i]–[ĩ]	[i]–[ĩ]	[i]–[iŋ]	[i]–[i]
[ĩ]–[iŋ]	[ĩ]–[iŋ]	[iŋ]–[iŋ]	[i]–[iŋ]

French listeners performed above chance on [ĩ]–[iŋ]; they can perceive nasality on a vowel, given their L1 grammar which contains non-high nasal vowel phonemes. Spanish listeners also performed above chance on [ĩ]–[iŋ], but for a different reason: the absence of [nasal] on vowels in their L1 grammar led to [ĩ]–[iŋ] being perceived as [i]–[iŋ]. English listeners did not perform above chance, revealing that their behaviour on the first contrast, [i]–[ĩ], was biased by perceptual illusion: they perceived an illusory nasal consonant following a nasal vowel and thus misidentified [ĩ] as [iŋ].

In the following lines, I couch perceptual illusion within a theory of licensing (Goldsmith, 1990; Itô, 1986). In L1 English, nasal vowel allophones can bear the feature [nasal] if and only if this feature is linked to and thus licensed by a following nasal consonant, which contrasts for the feature [nasal]; this is shown in (1a) for the word ‘sing’. It follows that, in an L2 setting, English listeners cannot perceive nasality on a vowel as distinct from a following nasal consonant. Consequently, in [iŋ], they cannot dissociate [nasal] from the nasal consonant and reanalyse it as an inherent property of the vowel, [ĩ], as in (1b). French listeners, in contrast, can redeploy [nasal], and build the representation that is ill-formed for English in (1b), because vowels, which contrast for the feature [nasal] in the L1 grammar of French, can license this feature. We return to this in the discussion.


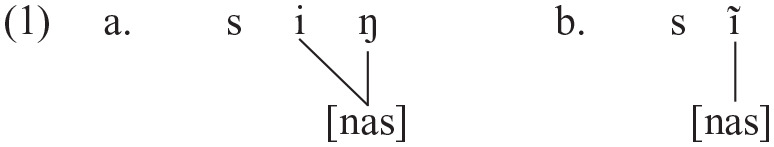


In sum, as only the French speakers succeeded in discriminating [i]–[ĩ], Martinez et al. conclude that feature redeployment at first exposure to a new contrast is only possible if the feature is contrastive in the L1 vowel system, even though nasality in both French and English extends far back into the vowel and thus should be readily detectable in the Portuguese stimuli based on listeners’ L1 experience. Perceptual success is thus being thwarted by the phonological status of the feature [nasal] in the L1 grammar: vowels that bear allophonic [nasal] cannot license this feature in English. Returning to Archibald’s defence of abstract symbolic representations, these results are consistent with a theory where the phonological status of a feature in the L1 grammar (contrastive or allophonic) affects naive listeners’ ability to discriminate new contrasts.

## III Why are some features unacquirable?

Thus far, we have seen that the status of a feature in the L1 grammar, as contrastive vs. allophonic, can shape L2 speech perception. We turn now to examine how L2ers’ perception is negatively impacted by the absence of a necessary feature from the L1 grammar. Although there is a large literature demonstrating that L2ers can acquire new contrasts, this finding does not extend to French-speaking learners’ experience with English [h], a segment that is lacking from the French grammar. It is incumbent on linguists who advocate for features to strive to explain why.

Francophones have difficulty distinguishing [h] from Ø (e.g. *hair* vs. *air*) and these difficulties are seemingly intractable. LaCharité and Prévost (1999) find that in an AX discrimination task, French-speaking L2ers perform significantly worse than English-speaking controls on [h] vs. Ø. This is surprising given that the French speakers under study had attained advanced proficiency in English, had studied phonetics, and were training to become English teachers. Janda and Auger (1992) report bidirectional substitutions between [h] and Ø in their study of spontaneous production data by French-speaking L2ers (e.g. *’ead[h]ache* for *headache*). Similar to LaCharité and Prévost, the participants in their study had attained advanced proficiency in English and had been living in English-speaking communities for several years.

The persistent difficulty that francophones experience with [h] may, in part, be due to its acoustic properties: [h] is a low intensity fricative with no constriction in the oral cavity, which could impede francophones’ ability to detect it in the speech stream. However, Mah (2011) and Mah et al. (2016) argue that this cannot be the whole story, as French speakers can perceive [h] when it is part of a non-linguistic speech stream; their difficulties are confined to contexts when [h] is part of a linguistic speech stream or when their stored representations for /h/-initial words are tapped. This suggests that a featural explanation holds.

Mah (2011) and Mah et al. (2016) probe the question of [h] perception using neuro-cognitive evidence from event-related potentials. In the first experiment, linguistic and non-linguistic stimuli were created, full syllables in the former case ([ʌm] ‘um’ vs. [hʌm] ‘hum’), and fricative noise bursts in the latter ([f] vs. [hf]). To assess participants’ detection of [h], they used the mismatch negativity (MMN; Näätänen, 2001), which is elicited when changes in stimuli cross the boundary between categories. French-speaking L2ers were observed to behave like the English controls in the non-linguistic condition: MMN components were elicited for both groups, indicating that listeners detected [h] as different from Ø when it was presented in fricative noise burst stimuli. However, the L2ers behaved differently from the controls in the linguistic condition: the MMN component was elicited only for the controls, indicating that only they were able to detect [h] as different from Ø when presented in full syllables.

This suggests that the francophones cannot build a phonological representation for [h], a conclusion that was further tested in an experiment that probed francophones’ stored representations for /h/-initial words. Mah (2011) designed a task where participants were presented with semantically infelicitous sentences, which have been shown in earlier work to elicit an N400 (Kutas and Hillyard, 1980). If participants have target-like stored representations for /h/-initial words like *hair*, that is, representations that are distinct from those for vowel-initial words like *air*, semantically infelicitous sentences such as Lots of girls want to have long shiny *air should elicit an N400. Mah (2011) finds that English-speaking controls behave as predicted: they showed a significant N400 response to the infelicitous sentences. Francophones, however, did not. This is as expected only if francophones cannot detect [h] in the speech stream.

Taken together, the results from these two experiments suggest that the perceptual problems that francophones have with [h] reflect the inability to perceive this sound as distinct from Ø. As the difficulties only manifest in linguistic tasks, and not with perceptual tasks more generally, this suggests that the problem lies with the inability to build and store an appropriate featural representation for English /h/. This would support Archibald’s claim that L2ers’ grammars employ abstract symbolic representations at the level of the segment.

An important question, though, remains unaddressed: why are francophones’ perceptual difficulties with [h] seemingly permanent? In the following paragraphs, I explore what might be behind this puzzling observation, starting with the types of voicing systems employed by each of the languages under focus.

Proponents of the theory of Laryngeal Realism argue that languages with a two-way laryngeal contrast on the VOT dimension represent this contrast in one of two ways: voicing lead vs. short lag languages employ the feature [voice], while short lag vs. long lag languages employ the feature [spread glottis] ([SG]) (e.g. Beckman et al., 2013; Honeybone, 2005). Table 2 shows that English employs [SG] and thus its aspirated stops bear this feature; French employs [voice] and thus its prevoiced stops bear the feature [voice]; finally, in both languages, short lag stops are unspecified for any laryngeal feature.

**Table 2.** Laryngeal features across languages.

	Prevoiced ([voice])	Short lag	Long lag / aspirated ([SG])	Type of *h*
English		/p/	/p^h^/	/h/
Dutch	/b/	/p/		/ɦ/
French	/b/	/p/		

Table 2 also suggests that there may be a correlation between the voicing feature that a language employs for stops and the type of *h* it has. In English, aspirated stops and /h/ have the same distribution: both are confined to prosodically strong positions, which Davis and Cho (2003) use to motivate an [SG] specification for /h/. As /h/ lacks independent supralaryngeal constriction (Keating, 1988), [SG] is the only feature that it underlyingly bears. Dialects of Dutch, a language with true voicing, employ /ɦ/, which involves vocal fold vibration, and thereby requires the feature [voice]. Unlike Dutch, French lacks *h* altogether; indeed, there are no consonants in the language without supralaryngeal features (*[h], *[ɦ], *[ʔ]). If perception is mediated by features, then with no [SG] in the L1 grammar of French, there is no feature for French-speaking learners to detect in English [h], thus resulting in perception failure (Mah, 2011).^2^

As mentioned, though, perceptual failure for French-speaking L2ers of English [h] seems to be permanent. Increased sensitivity to the acoustic properties of [h], which might be expected with increased exposure to English, is seemingly not observed. The explanation for this may lie in the representation of voicing in English and French. English [h] would presumably be acquirable if French stop contrasts employed the feature [SG]. There is, though, no ‘incentive’ for learners to project [SG]. Both languages can capture a two-way laryngeal contrast for stops; the laryngeal contrast in French speakers’ L2 English can thus be effectively, although incorrectly, represented using the L1 feature [voice]. While some French-speaking L2ers of English do produce aspiration in English, suggesting that they attend to cues like the release bursts present on aspirated stops, this may lead them to shift their VOT boundary without amending their featural representations. As Mah (2011) points out, consistent with this, Caramazza et al. (1973) and Flege (1987) observe that the VOT values of French-speaking L2ers for English stops are intermediate between those of English and French monolinguals. Additional evidence for a shift comes from over-aspiration; Swanson (2006) and Mah (2011) find that some L2ers who aspirate also produce aspiration in sC clusters (e.g. *s[t^h^]op*), a context where it is not found in English because the wide glottis associated with [SG] has sufficiently narrowed throughout the duration of the fricative (Iverson and Salmons, 1995). We return to the significance of these findings for a featural account of misperception in the discussion.

In sum, French-speaking L2ers have been shown to detect [h] in a non-linguistic task, but fail to detect it or reliably produce it in linguistic tasks. This suggests that learners can hear [h] but that they cannot build a featural representation for it. This is consistent with Archibald’s claim that abstract symbolic representations, namely features, underlie L2ers’ phonological behaviour.

## IV Discussion and conclusions

In this commentary, I set out to test whether there is evidence for Archibald’s claim that abstract symbolic representations regulate L2 behaviour. I strived to answer this question in the affirmative by showing that L2 perception is, in part, mediated by features. Two studies were reported on in support of Archibald’s claim. The first study (Martinez et al., 2023) showed that the status of the feature [nasal] in vowels in the L1 grammar, as contrastive (French) or allophonic (English), can impact L2 perception of nasal vowels in the L2 (Brazilian Portuguese). Although nasality in French and English extends far back into the vowel and thus should be readily perceptible based on L1 experience for L2ers from both languages, perceptual success for English speakers was hindered by the phonological status of [nasal] in the L1 grammar: vowels that bear allophonic [nasal] in English cannot license this feature. Consequently, [nasal] cannot be detached from and perceived independently of a following nasal consonant. This supports a role for abstract phonological representations: features; structures that can formally express the presence or absence of feature sharing; and a theory of feature licensing that can capture in a principled way the licensing potential of different prosodic positions (Harris, 1997).

The second study (Mah, 2011; Mah et al., 2016) showed that the absence of the feature [SG] from the L1 grammar of French can negatively impact the ability to perceive and build novel /h/ in the L2 grammar that francophones construct for English. French speakers are able to perceive [h] in non-linguistic stimuli, but fail to perceive it in linguistic stimuli, which suggests that they do not lack the ability to hear [h] but, rather, to build a featural representation for this segment. Although L2ers could potentially be successful if they were to acquire [SG] for English stops, there is seemingly no motivation to do this: they can use their L1 feature [voice] to capture the English laryngeal contrast, if they shift their VOT boundary. The ability to shift the boundary suggests that L2ers can make use of phonetic cues like the release bursts present on aspirated stops to approximate target-like production of English aspiration; however, without [SG], their VOT values fall between those of native L1 (French) and target L2 (English); they overapply aspiration to stops in sC clusters; and they fail to perceive [h]. In short, the lack of the necessary [SG] feature has consequences beyond the inability to discriminate [h] from Ø. This is as expected from a phonological account, as features have a classificatory function, which means that they are expected to shape phonological behaviour across multiple groups of segments.

The results we have presented serve to endorse Archibald’s position that abstract symbolic representations regulate (L2) phonological patterning. The results are consistent with a theory that advocates for features and shows that their phonological status (contrastive or allophonic; present or absent) can impact L2ers’ ability to discriminate new contrasts. At the same time, it is important to point out that a feature-based approach does not deny a role for phonetic cues in perception (see, for example, Archibald, 2023). Instead, features can skew how L2ers interpret these cues, thereby facilitating or hindering the establishment of new contrasts.

## Notes

1. The term contrast is used differently by different researchers. Here, I use it in its narrowest sense: a feature is contrastive if it is needed to establish the phonemic inventory of a language. Contrastive can thus be equated with phonemic and juxtaposed with allophonic (for thorough discussion of this issue, see Dresher, 2009; Hall, 2009).

2. In English, /h/ tends to be realized as voiceless [h] in domain-initial position and as voiced [ɦ] in other positions (Garellek et al., 2023). It is conceivable that French speakers could redeploy [voice] to facilitate perception of [ɦ] in words like *ahead* and *vehicular*.
